# *Osmoxylon*‐like fossils from early Eocene South America: West Gondwana–Malesia connections in Araliaceae

**DOI:** 10.1002/ajb2.70045

**Published:** 2025-05-19

**Authors:** Peter Wilf

**Affiliations:** ^1^ Department of Geosciences and Earth and Environmental Systems Institute Pennsylvania State University, University Park 16802 PA USA; ^2^ IUCN/SSC Global Tree Specialist Group, Botanic Gardens Conservation International Richmond TW9 3BW UK

**Keywords:** Araliaceae, biogeography, fossil rainforests, Laguna del Hunco, leaf architecture, Malesia, *Osmoxylon*, paleoconservation, Patagonia

## Abstract

**Premise:**

Araliaceae comprise a moderately diverse, predominantly tropical angiosperm family with a limited fossil record. Gondwanan history of Araliaceae is hypothesized in the literature, but no fossils have previously been reported from the former supercontinent.

**Methods:**

I describe large (to macrophyll size), palmately compound‐lobed leaf fossils and an isolated umbellate infructescence from the early Eocene (52 Ma), late‐Gondwanan paleorainforest flora at Laguna del Hunco in Argentine Patagonia.

**Results:**

The leaf fossils are assigned to *Caffapanax canessae* gen. et sp. nov. (Araliaceae). Comparable living species belong to five genera that are primarily distributed from Malesia to South China. The most similar genus is *Osmoxylon*, which is centered in east Malesia and includes numerous threatened species. The infructescence is assigned to *Davidsaralia christophae* gen. et sp. nov. (Araliaceae) and is also comparable to *Osmoxylon*.

**Conclusions:**

The *Caffapanax* leaves and *Davidsaralia* infructescence, potentially representing the same source taxon, are the oldest araliaceous macrofossils and provide direct evidence of Gondwanan history in the family. The new fossils and their large leaves enrich the well‐established biogeographic and climatic affinities of the fossil assemblage with imperiled Indo‐Pacific, everwet tropical rainforests. The fossils most likely represent shrubs or small trees, adding to the rich record of understory vegetation recovered from Laguna del Hunco.

The Araliaceae (Apiales), best known for ivies, ginsengs, and other ornamental and medicinal plants, consist of 46 genera and ca. 2000 species of herbs, shrubs, and trees (Lowry and Plunkett, 2024). The family contributes significantly to the structure and biodiversity of tropical forest understories and has some temperate representatives (Plunkett et al., 2018; Lowry and Plunkett, 2024). Araliaceae are closely related to the mainly herbaceous Apiaceae (Plunkett et al., 1996; Wen et al., 2021) and have a long history of study (for historical bibliographies, see van Steenis, 1955; Frodin and Govaerts, 2003). They have frequently been the focus of studies on evolutionary timing and biogeography, especially North America–Asia disjunctions (Wen et al., 1998; Wen, 1998, 2000; Nicolas and Plunkett, 2014; Zuo et al., 2017; Valcárcel and Wen, 2019). However, early (pre‐Neogene) Araliaceae macrofossils are few and probably limited to Eocene North America (Manchester, 1994; Manchester et al., 2015, 2024). Various authors have suggested that the family had a history in Gondwana (Raven and Axelrod, 1974; Philipson, 1979; Plunkett et al., 2004; Mitchell et al., 2012; Nicolas and Plunkett, 2014; Valcárcel et al., 2014) without direct fossil evidence from the former supercontinent.

The Laguna del Hunco fossil site in northwest Chubut, Patagonian Argentina, preserves early Eocene (ca. 52 Ma), late Gondwanan, highly fossiliferous caldera‐lake beds (Berry, 1925; Petersen, 1946; Aragón and Mazzoni, 1997; Wilf et al., 2003; Gosses et al., 2021; Hajek et al., 2025). Plant macrofossils from Laguna del Hunco primarily comprise compressions, complemented by a diverse palynoflora (Barreda et al., 2020; Zamaloa et al., 2006, 2020) and a suite of silicified trunks (Bomfleur and Escapa, 2019; Brea et al., 2021; Pujana et al., 2020, 2025). The fossil flora has been heavily sampled (>8000 macrofossil specimens) and well studied over the past 25 years through an ongoing Argentine–U.S. collaboration based at the Museo Paleontológico Egidio Feruglio (MEF, Trelew, Argentina). The assemblage ranks among the most diverse for the Eocene globally, with more than 185 species (based on leaf species and morphotypes) and over 30 plant families (Barreda et al., 2020; Wilf et al., 2003, 2005a, 2024).

The Laguna del Hunco flora is probably best known for its numerous occurrences of plant taxa whose extant relatives survive at great distance in Australasia and Malesia, via Gondwanan connections to Australia through Antarctica and the Neogene Australia–Asia collision (Romero and Hickey, 1976; Zamaloa et al., 2006; Gandolfo et al., 2011; Wilf, 2012; Carvalho et al., 2013; Carpenter et al., 2014; Wilf and Escapa 2015, 2016; Gandolfo and Hermsen, 2017; Rossetto‐Harris et al., 2020; Zamaloa et al., 2020; Brea et al., 2021; Matel et al., 2022; Andruchow‐Colombo et al., 2019, 2023; Wilf et al., 2009, 2014, 2019, 2023, 2024). Notably, the diverse insect‐herbivory traces observed on fossil *Agathis* and *Eucalyptus* leaves from Laguna del Hunco persist on their Old World living relatives (Donovan et al., 2020, 2023; Giraldo et al., 2025). In their modern ranges, many species of the “survivor” genera are severely threatened by climate change and land clearing (Kooyman et al., 2014, 2022; Wilf and Kooyman, 2023, 2025).

Here, I report rare (16 specimens), often large, palmately lobed leaves and a unique, isolated umbellate infructescence from Laguna del Hunco as new taxa of Araliaceae. Palmately lobed leaves occur in many angiosperm groups, but additional features of the fossils allow all but Araliaceae to be eliminated as likely relatives. Within Araliaceae, only five genera, all with significant Indo‐Pacific ranges, are comparable, especially *Osmoxylon*, which is centered in east Malesia (Philipson, 1976; Utteridge and Frodin, 2021). The infructescence is also similar to a lateral umbellule of *Osmoxylon*. I describe these new taxa and discuss their evolutionary, biogeographic, paleoenvironmental, and conservation significance.

## MATERIALS AND METHODS

The fossils reported here were collected during several MEF expeditions from 1999 to 2019 at quarries LH04, LH13, LH15, LH20, LH23, LH27, and LH29 in the composite stratigraphic section of the Tufolitas Laguna del Hunco at Laguna del Hunco (Wilf et al., 2003; Hajek et al., 2025). The section is exposed around the small modern playa known as Laguna del Hunco (Lake of Rushes), near S42.5°, W70.0° in the Piedra Parada Caldera complex of northwestern Chubut Province, Argentina (Petersen, 1946; Aragón and Mazzoni, 1997; Gosses et al., 2021). The early Eocene paleolatitude of the strata was approximately 47° S (Wilf et al., 2005a; Hajek et al., 2025). The complete stratigraphic log (Wilf et al., 2003) with coordinates and stratigraphic positions for all macrofossil quarry locations (LH01–LH33) was recently revised (Hajek et al., 2025). The ages of all fossil occurrences at Laguna del Hunco are constrained to a ca. 200 kyr (52.2–52.0 million years ago [Ma]) window of the early Eocene (Ypresian) using several ^40^Ar‐^39^Ar and U‐Pb TIMS analyses of volcanic minerals from airfall tuffs (Wilf et al., 2003, 2005a; Gosses et al., 2021; Hajek et al., 2025). Specifically, recent U‐Pb TIMS ages on zircons of 52.217 ± 0.014 Ma and 51.988 ± 0.035 Ma from the lower and upper portions, respectively, of the Tufolitas at Laguna del Hunco directly bracket all the fossil localities (Hajek et al., 2025). An additional 52.153 ± 0.013 Ma U‐Pb age from the middle of the most fossiliferous interval (Hajek et al., 2025) validated the previously widely cited ^40^Ar‐^39^Ar age of 52.22 ± 0.22 Ma analyzed from the same sample and further constrains nearly all fossils described here (except those from LH27, which sits above) to a ca. 70 Kyr interval, namely ca. 52.217–52.153 Ma.

The Tufolitas Laguna del Hunco formed through lacustrine fill, beginning no earlier than 52.54 ± 0.17 Ma (Gosses et al., 2021), of the crater depression resulting from the massive Eocene eruption of the Piedra Parada Caldera (Aragón and Mazzoni, 1997; Aragón et al., 2018). Several publications have described the geology of the Piedra Parada Caldera and the embedded Tufolitas Laguna del Hunco (Petersen, 1946; Aragón and Mazzoni, 1997; Aragón et al., 2001, 2004, 2018; Gosses et al., 2021; Hajek et al., 2025). From detailed facies analyses, Hajek et al. (2025) concluded that slope failures of the surrounding steep, wet caldera walls rapidly moved plant material to the lake bottom, leading to the exceptional fossil preservation at Laguna del Hunco. The paleoenvironment is well understood as an everwet (aseasonal), frequently disturbed rainforest, based on the modern habitats and physiological requirements of the flora's living relatives; fossil plant tissues closely tied to rainforest habitats, including transfusion tissues in podocarp conifer leaves and vessel‐less wood of fossil Winteraceae; and the presence of plants whose living relatives are disturbance specialists (*Eucalyptus*, *Macaranga*; Gandolfo et al., 2011; Wilf, 2012; Brea et al., 2021; Andruchow‐Colombo et al., 2023; Wilf et al., 2009, 2023). The most comparable extant climates, edaphic settings, and floristic composition occur in east Malesian lower montane zones, especially in New Guinea (Wilf et al., 2009, 2019, 2023; Kooyman et al., 2014; Brea et al., 2021; Wilf and Kooyman, 2023; Hajek et al., 2025).

Macrophotography at MEF utilized a series of DSLR cameras over several years; most images used here were taken on Canon EOS 7D and Nikon D850 DSLRs (Canon, Tokyo, Japan; Nikon, Melville, NY, USA). Microphotography of the infructescence fossil was done at MEF using a Nikon Eclipse 50i compound microscope with a Nikon DSFi3 camera and a DS‐L4 control pad. Three‐dimensional scanning was not attempted because of the highly flattened, coalified preservation. All fossil materials reported here are housed in the MEF Paleobotany Collections (Trelew, Argentina), abbreviated MPEF‐Pb (letter suffixes in the figure captions indicate specific parts and counterparts).

Herbarium comparisons were done using specimens from the United States National Herbarium (US; Washington, D.C.) and digital collections. Many Araliaceae species are poorly vouchered because their large leaves and inflorescences exceed the standard herbarium sheet size (Philipson, 1979). To address this issue, I consulted the literature and inspected herbarium sheets to search for smaller and more complete specimens. The considerable number of online voucher images that are now available from the world's herbaria (Davis, 2023) provided an outstanding resource.

The online image collections used here included version 2.0 of an open‐access data set of cleared and x‐rayed leaves (Wilf et al., 2021) (https://doi.org/10.25452/figshare.plus.14980698.v2) and several online herbaria. The most accessed institutions for this study were the Naturalis Biodiversity Center, Leiden (L; https://bioportal.naturalis.nl); US (https://collections.nmnh.si.edu/search/botany); Herbarium of the Arnold Arboretum, Harvard University Herbaria (A; https://kiki.huh.harvard.edu/databases/specimen_index.html); and Herbier National, Muséum National d'Histoire Naturelle, Paris (P; https://science.mnhn.fr/institution/mnhn/collection/p/item/search/form). Aggregator sites included the Global Biodiversity Information Facility (GBIF; https://www.gbif.org), the Chinese Virtual Herbarium (https://www.cvh.ac.cn), and JSTOR Global Plants (https://plants.jstor.org), which is particularly valuable for its emphasis on type specimens. However, several other online herbaria provided important virtual specimens. All herbarium material figured or cited here is referenced using the standard institutional codes from Index Herbariorum (Thiers, updated continuously). Live *Osmoxylon novoguineense* plants were photographed at the Fairchild Tropical Botanic Garden (Miami, FL, USA; see Acknowledgments).

General taxonomic references consulted included the Araliaceae Central Website (Lowry and Plunkett, 2024) and works by Philipson (1979), Frodin and Govaerts (2003), Qibai and Lowry (2007), and Plunkett et al. (2018), with additional sources cited throughout the text. Except when necessary to circumscribe new taxa, taxon authorities are omitted in the text to increase readability. Nomenclature and diversity data follow Kew Plants of the World Online (https://powo.science.kew.org) and Araliaceae Central (Lowry and Plunkett, 2024). Authorities for plant fossils mentioned can be found in the respective cited publications. Leaf‐architecture terminology follows that of Ellis et al. (2009). Previously published scores for insect‐feeding damage type (DT) occurrences on fossil leaves (Wilf et al., 2005b) were revised using the standard categorization system (Labandeira et al., 2007).

## RESULTS

### Systematics

***Family*** **—**Araliaceae Juss.

***Genus*** **—** *Caffapanax* Wilf gen. nov.

***Generic diagnosis*** **—**As for the type species, due to monotypy.

**Figure 1:**
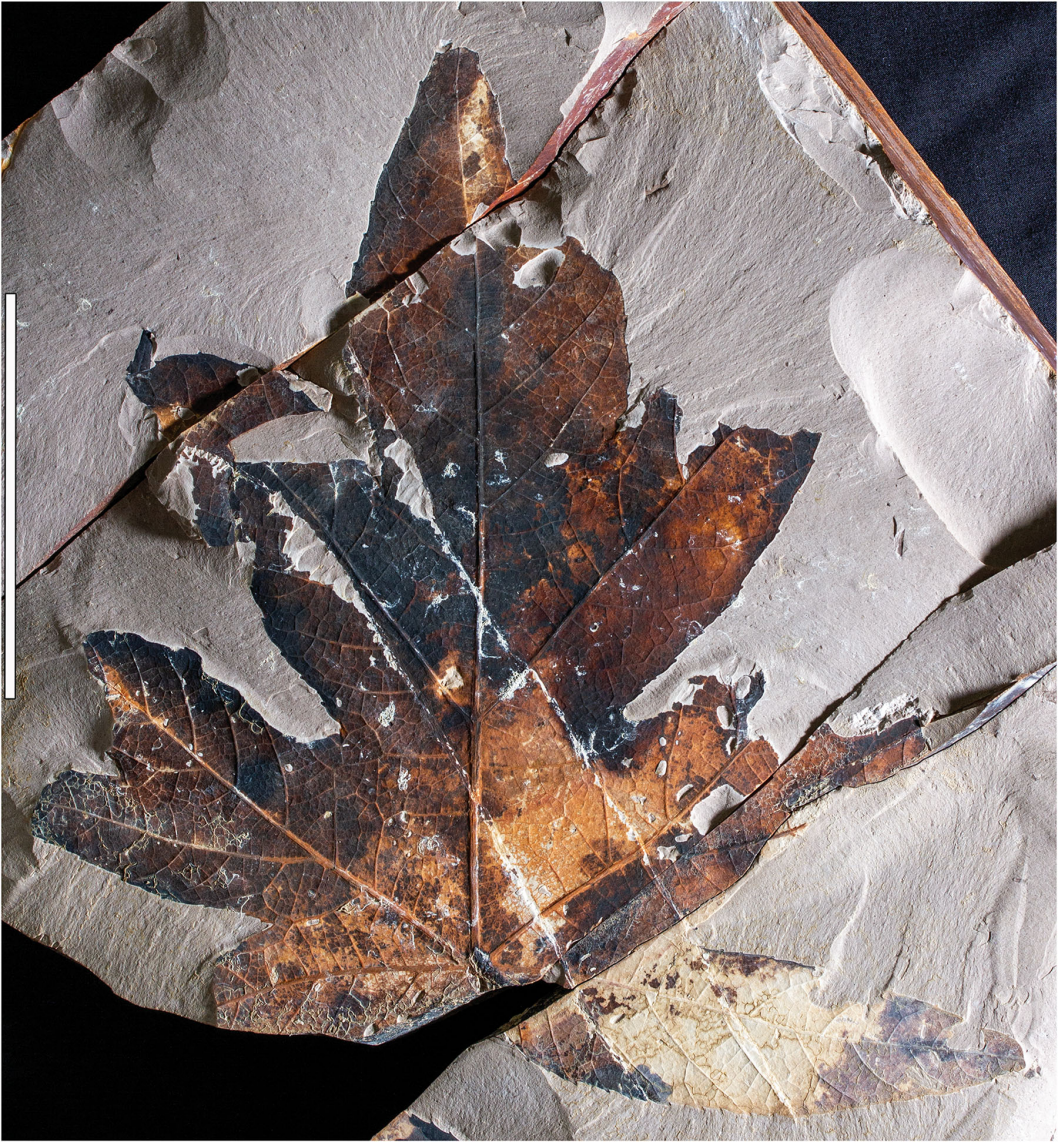
*Caffapanax canessae* gen. et sp. nov., holotype, MPEF‐Pb 10171a (see Figure 2A for counterpart). Specimen has five primary lobes, including a pair of reflexed basal lobes, a pair of lateral lobes each with one sublobe, and an expanded medial lobe with paired sublobes. Scale bar: 10 cm.

**Figure 2:**
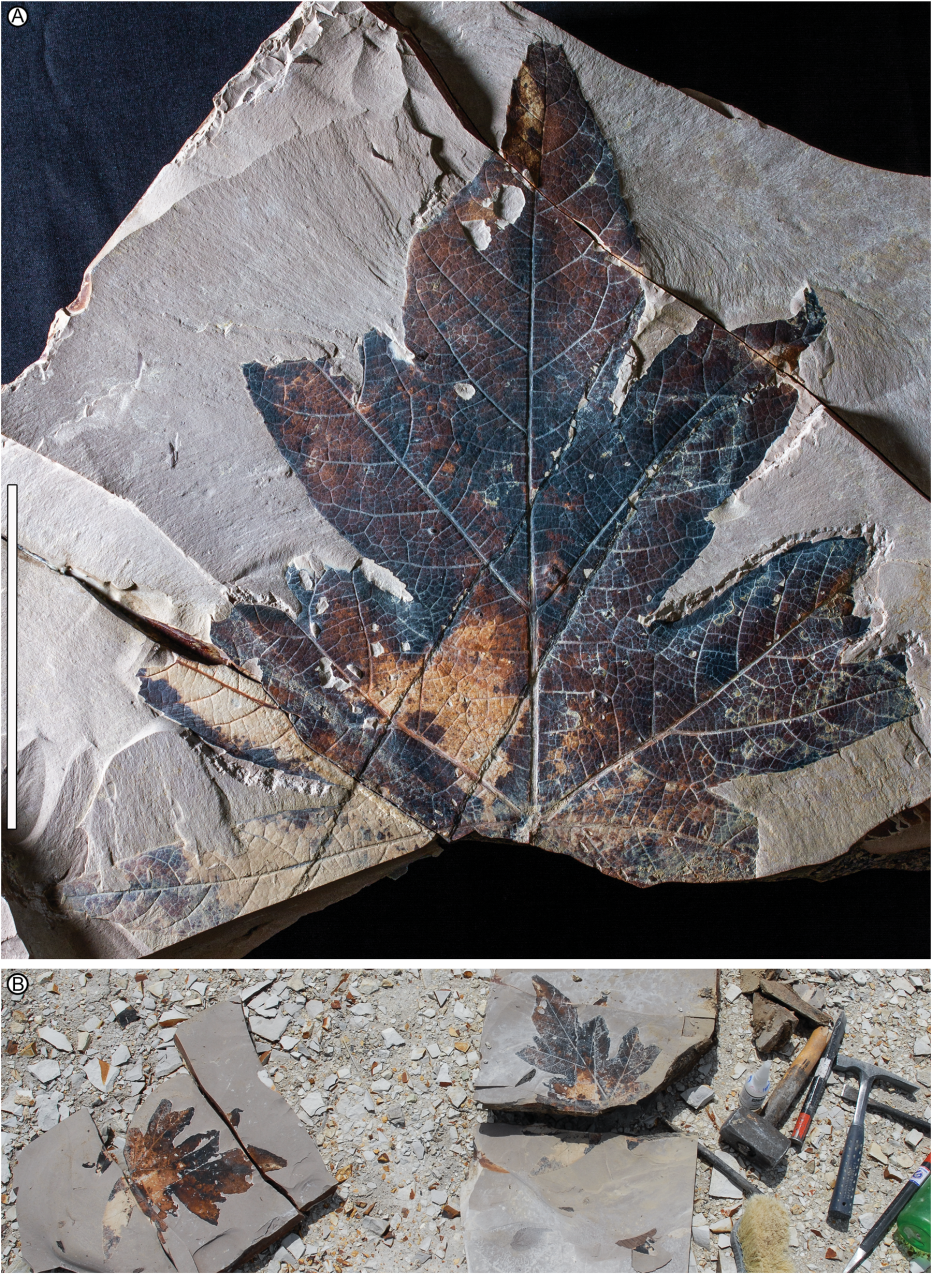
*Caffapanax canessae* gen. et sp. nov., holotype. (A) MPEF‐Pb 10171b (counterpart, see Figure 1 for part). Scale bar: 10 cm. (B) Discovery photo with part and counterpart, Laguna del Hunco quarry LH04, 21 November 2006.

**Figure 3:**
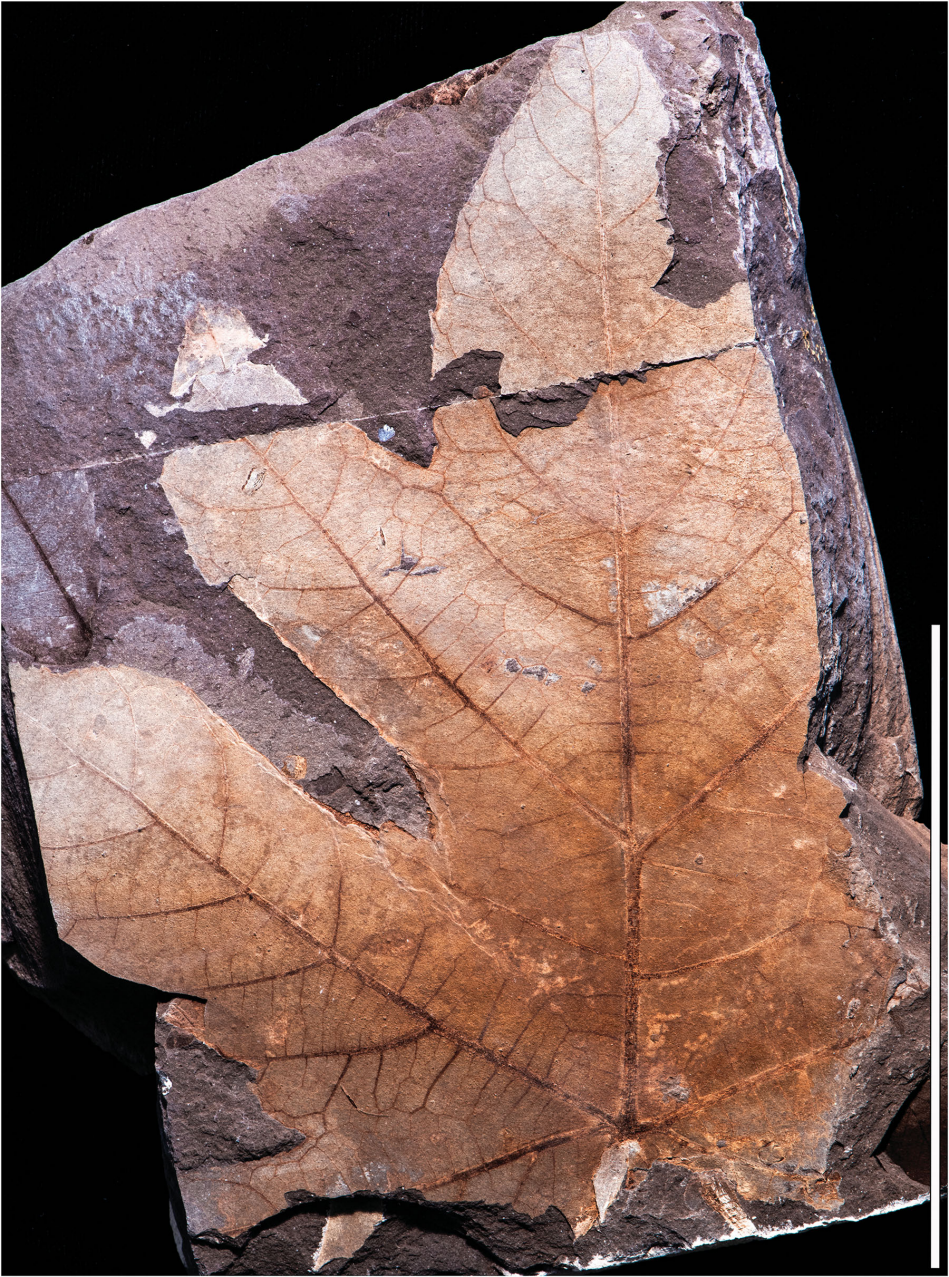
*Caffapanax canessae* gen. et sp. nov., paratype MPEF‐Pb 10182a. Specimen similar in form to the holotype (Figures 1, 2). Presence of lateral sublobes indeterminate due to preservation. Scale bar: 10 cm.

**Figure 4:**
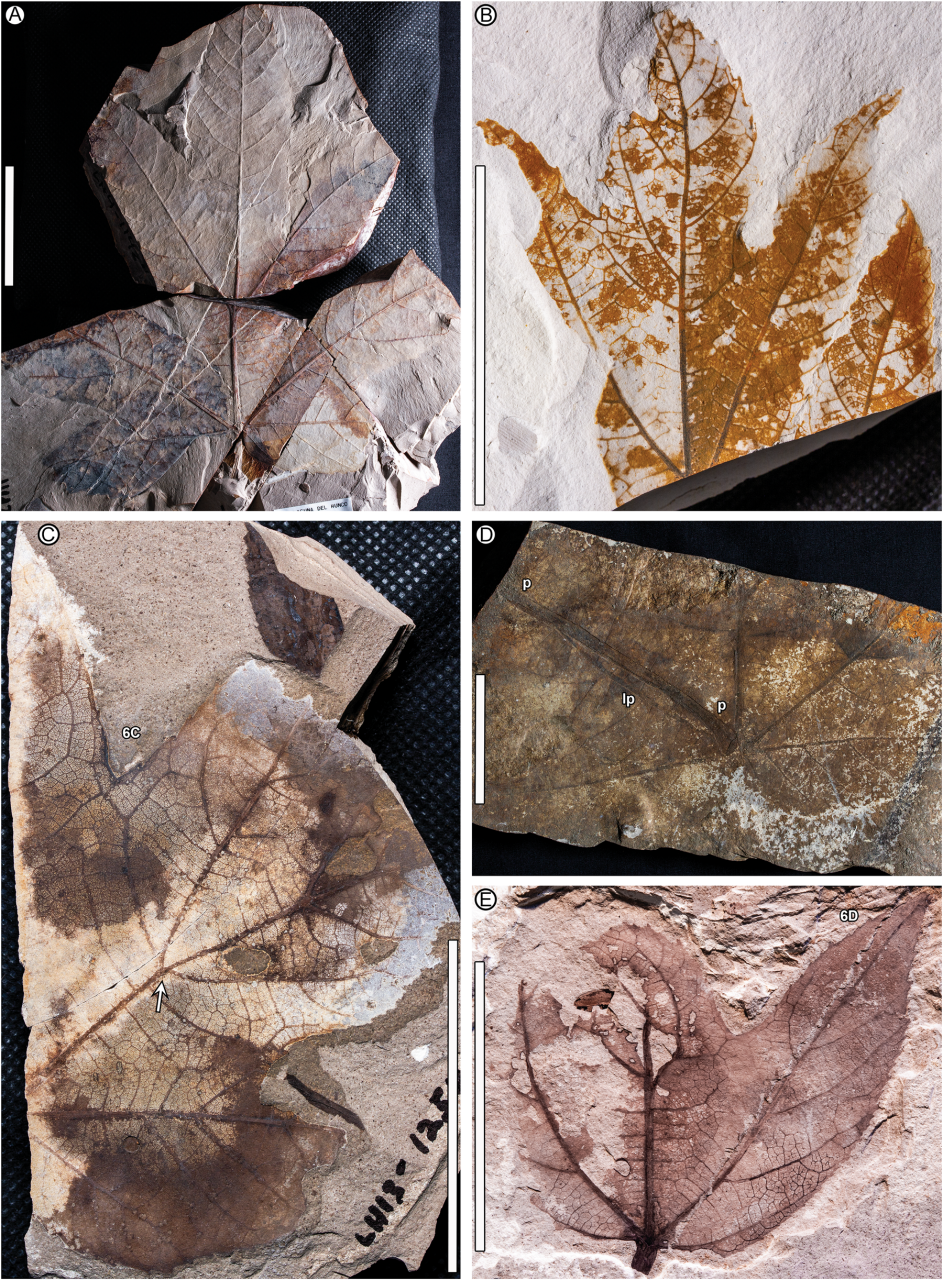
*Caffapanax canessae* gen. et sp. nov., showing variation in leaf form and preservation. (A) Paratype MPEF‐Pb 10170, similar in form to the holotype and other paratype (Figures 1, 2, 3). (B) MPEF‐Pb 1596, preserving a medial pinnately compound sublobe (cf. Figures 1, 2, 3) and the apex of a lateral lobe (lower right); this fossil can be easily mistaken for a pinnately lobed leaf. (C) MPEF‐Pb 10183, preserving most of one longitudinal half of the blade but without the midvein; the lateral lobe is divided. Arrow, divergence of lobe and sublobe midveins. Text label, location of detail in Figure 6C. (D) MPEF‐Pb 10178, poorly preserved leaf base with large petiole remnant (below the “p” labels) flattened and incompletely exposed under the blade, abruptly increasing in width at insertion. Note the much greater width of the petiole compared with the subjacent lateral primary vein (lp). (E) MPEF‐Pb 10175, three‐lobed form (see also Figure 6D) with petiole remnant, two damaged lobe apices, and one intact lateral lobe (at right), hole feeding along a secondary vein (DT50, at distalmost preserved blade portion), and other hole and margin external feeding (DTs 3, 14, 15) including the complete excision of the medial lobe apex. Text label, location of detail in Figure 6D. Scale bars: 5 cm (all panels).

**Figure 5:**
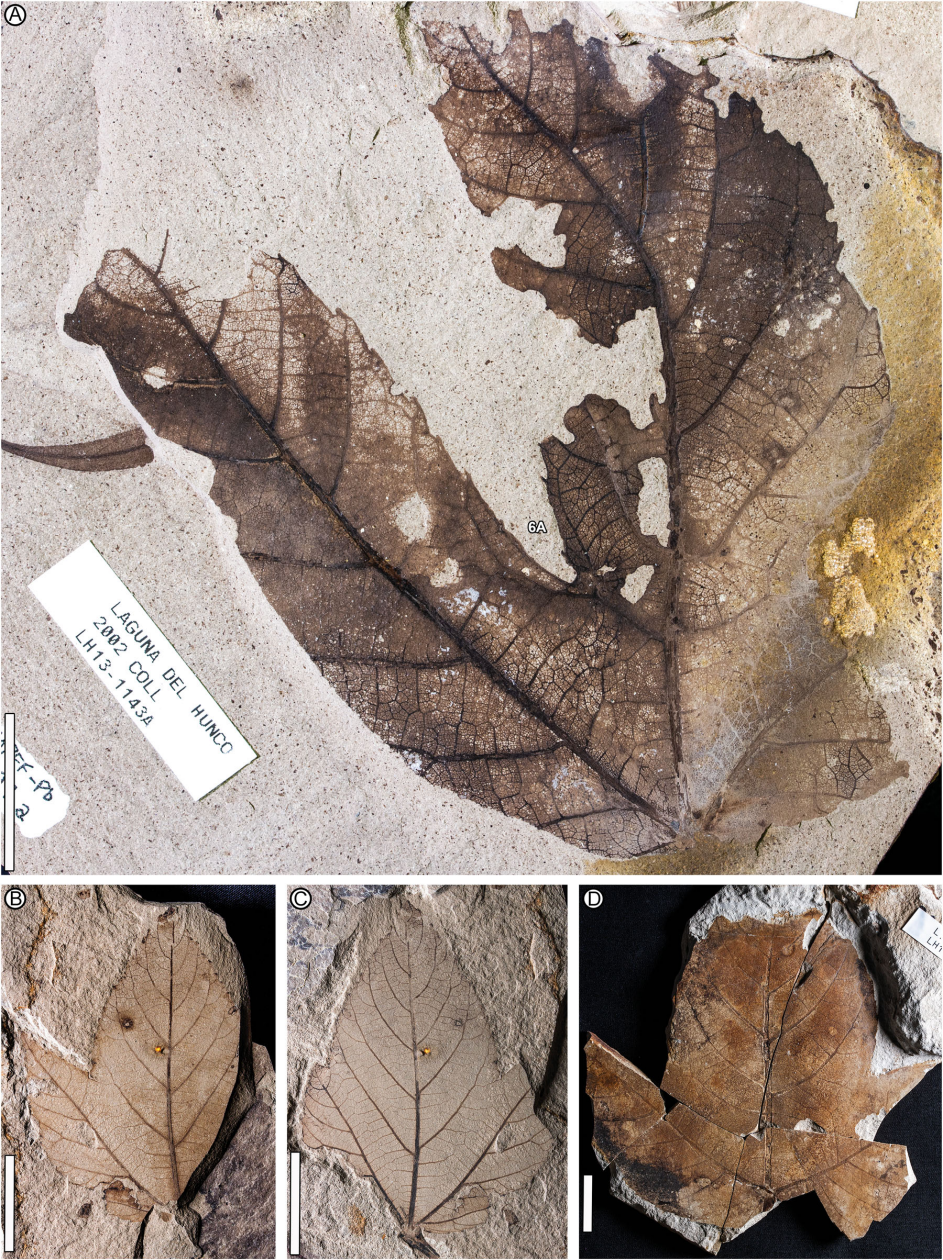
*Caffapanax canessae* gen. et sp. nov., forms with three undivided primary lobes (see also Figure 4E). (A) MPEF‐Pb 1597a, with excellent preservation of fine venation and margin portions; medial lobe narrow, simple, with significant insect‐chewing damage on its left margin (DTs 13–15). Text label, location of counterpart (mirrored) detail in Figure 6A. (B, C) MPEF‐Pb 10181b (B) and MPEF‐Pb 10181a (C, with petiole remnant), with unequal lateral lobe development and a simple, expanded medial lobe. Circular lesions from possible galls or fungal growths are visible on the left side (in the view of B, or the right in A) of the medial lobe. (D) MPEF‐Pb 10177; medial lobe expanded, simple. Scale bars: 2 cm (all panels).

**Figure 6:**
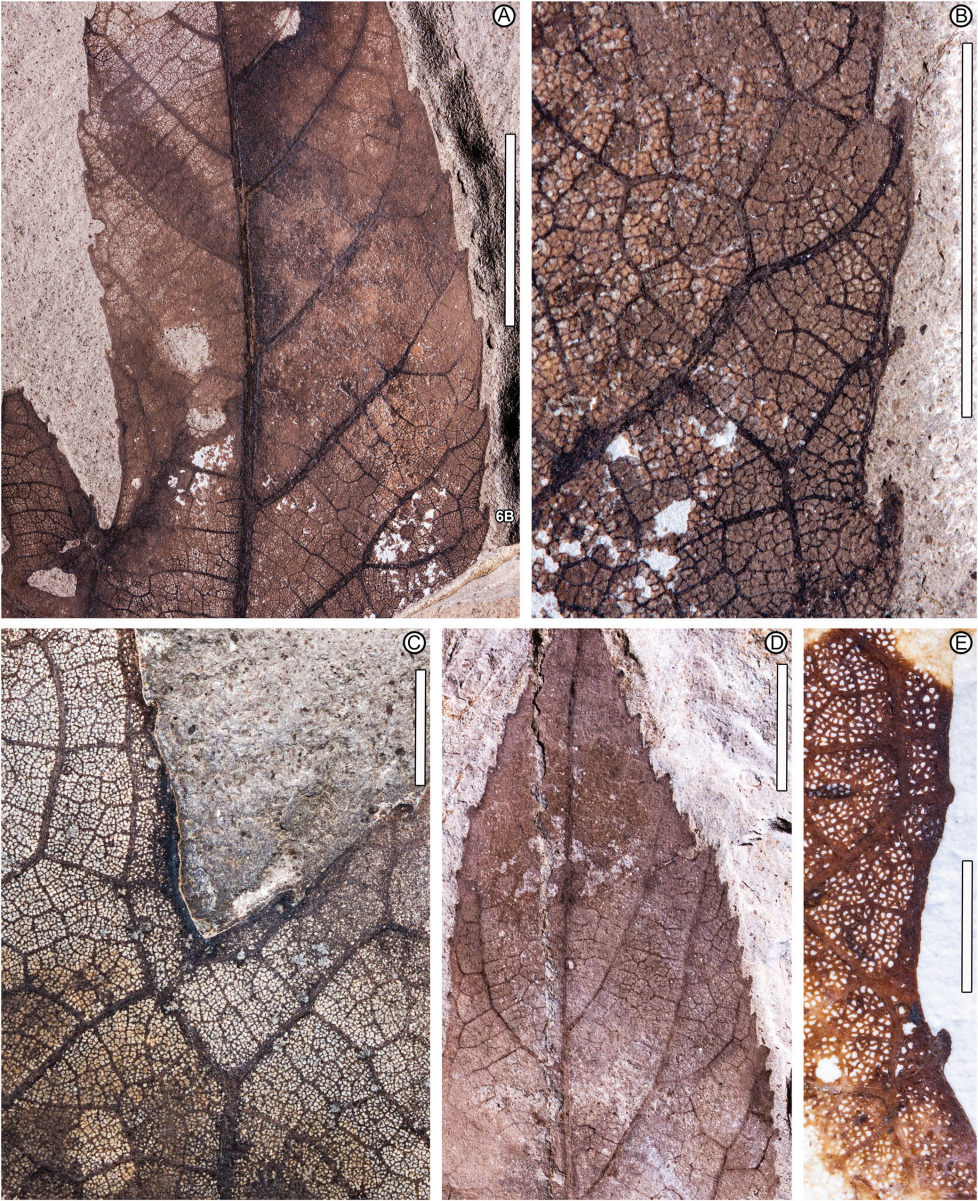
*Caffapanax canessae* gen. et sp. nov., details of narrow, small but variably sized and spaced teeth, fine‐reticulate areolation, and fimbrial veins. (A, B) MPEF‐Pb 1597b (counterpart of part in Figure 5A). Text label, location of detail in (B). (C) MPEF‐Pb 10183 (see Figure 4C). (D) MPEF‐Pb 10175 (see also Figure 4E). (E) MPEF‐Pb 10172a. Scale bars: 1 cm (A, D); 5 mm (B, C, E).

***Type species—**Caffapanax canessae* Wilf sp. nov. (Figures 1, 2, 3, 4, 5, 6).

***Holotype*** **—**MPEF‐Pb 10171 (Figures 1, 2), Laguna del Hunco, Tufolitas Laguna del Hunco of the Huitrera Formation, early Eocene (Ypresian). Collected at quarry LH04 (Wilf et al., 2003; Hajek et al., 2025), 21 November 2006. Repository: Museo Paleontológico Egidio Feruglio Paleobotany Collection (MPEF‐Pb), Trelew, Chubut, Argentina.

***Paratypes*** **—**MPEF‐Pb 10182 (Figure 3), collected at quarry LH29, 22 November 2019, and MPEF‐Pb 10170 (Figure 4A), collected at quarry LH04, 3 December 2002. Both specimens from Laguna del Hunco, Tufolitas Laguna del Hunco of the Huitrera Formation, early Eocene (Ypresian). Repository: Museo Paleontológico Egidio Feruglio Paleobotany Collection (MPEF‐Pb), Trelew, Chubut, Argentina.

***Additional specimens (13)*** **—**From quarry LH04: MPEF‐Pb 10172 (Figure 6E). From LH13: MPEF‐Pb 1595, 1597 (Figures 5A, 6A, B), 10173, 10174, 10175 (Figures 4E, 6D), 10183 (Figures 4C, 6C). From LH15: MPEF‐Pb 10176, 10177 (Figure 5D). From LH20: MPEF‐Pb 10178 (Figure 4D). From LH23: MPEF‐Pb 10179. From LH27: MPEF‐Pb 10181 (Figure 5B, C). From a float slab: MPEF‐Pb‐1596 (Figure 4B). All are from the Tufolitas Laguna del Hunco, Huitrera Formation, early Eocene (Ypresian). Updated maps, coordinates, and stratigraphic positions for all quarries are given by Hajek et al. (2025).

***Previous reference—*** Morphotype TY106, “unknown dicot sp.” (Wilf et al., 2005a: table A2; Merkhofer et al., 2015).

***Etymology—*** The new generic and specific epithets honor Técnicos Mariano Caffa and Leandro Canessa, respectively, of the MEF for their admirable record over more than two decades of fieldwork and fossil discovery in Patagonia. They continue to make significant contributions to paleontology, including the holotype of the new species (Figures 1, 2) unearthed by Téc. Caffa.

***Diagnosis*** **—**Petiole insertion marginal; base cordate. Blade size microphyll to macrophyll; length:width (L:W) ratio near or below 1:1. Primary veins basally actinodromous, regular, robust. Blade palmately lobed with three or five wide, convex primary lobes; lobe size increases markedly distally. When blade is five‐lobed, the basal lobes reflexed, the next lateral lobe pair undivided or with one sublobe per lobe, and the medial lobe much larger than the laterals, with a prominent pair of pinnate sublobes. Margin serrate; teeth short, narrow, non‐glandular. Major and minor secondary veins craspedodromous, terminating as tooth principal veins; fimbrial vein present. Higher‐order venation well organized; areolation impressed in tiny quadrangular to pentagonal fields; freely ending veinlets absent.

***Specific description*** **—** *Petiole* inserts marginally; petiole length more than 113 mm, petiole base and full length not preserved; petiole width 2.1–5.2 mm, expanding to 4.6–10 mm at insertion (Figures 4D, 4E, 5C). The *lamina* has a cordate base and serrate margin, spines absent, L:W ratio near 1:1 (0.7–1.1:1), length to 230 mm, width to 280 mm, size categories range from microphyll (Figure 5B,C) to macrophyll (Figures 1, 2, 3, 4A), symmetrical except in smaller leaves with uneven lateral lobe development (Figure 5B,C), and palmately lobed with 3–5 convex primary lobes, the lobe sinuses rounded, the lobe apices narrow‐acute. *Lobe* size markedly increases distally, especially in larger leaves (Figures 1, 2, 3A, B), which are five‐lobed, including a pair of small, reflexed basal lobes with 90°–110° divergence from the midvein. The pair of lateral lobes in five‐lobed leaves is larger than the basal pair, with a divergence of 50°–60° (40°–60° in three‐lobed leaves), and is undivided or with one sublobe per lobe (Figures 1, 2, 4C). Lateral lobe incision to the midvein (along the distal lobe flank) is approximately 70%–80%. The medial lobe in the five‐lobed leaves is abruptly larger than the laterals, with a pair of prominent, wide, pinnate sublobes diverging at 35°–50°, estimated incision 50%–60%.

Primary *venation* is basal actinodromous with 3–5 thick, regular primary veins corresponding to the primary lobes; simple agrophic veins are variably present, mostly on three‐lobed leaves. Major secondaries are excurrent or decurrent and craspedodromous; spacing increases proximally; angle is slightly irregular. Intersecondaries are absent; interior secondaries are present; minor secondary course is craspedodromous. Fimbrial vein is present (Figure 6). Intercostal tertiary veins are mixed percurrent to irregular reticulate, course straight to convex, angle to primary obtuse and consistent or decreasing exmedially. Epimedial tertiaries are mixed percurrent, proximal course perpendicular to the primary, distal course basiflexed and parallel to intercostal tertiaries. The quaternary vein fabric is random reticulate, and the quinternary vein fabric is regular reticulate, forming small, quadrangular, well‐developed areoles (Figure 6B, C, E). Freely ending veinlets are absent.

*Teeth* (Figure 6) are diminutive, though variably sized, and narrow, spacing 1–3(4) teeth/cm, usually in one order. Tooth sinuses are shallow and rounded; tooth shapes are concave/convex and straight/convex. Major and minor secondaries become the tooth principal veins, which along with the fimbrial vein fill much of the limited tooth area. The principal vein is deflected at tooth entry and terminates, with or without a slight expansion, at the nonglandular tooth apex. The dark appearance of many teeth results from compression of the relatively large principal and fimbrial veins in the small tooth area, rather than from glands, as can be seen in the best‐preserved teeth (Figure 6). More than half (9 of 16) of the leaves have sparse insect‐feeding damage, primarily from external feeding (hole, surface, and margin feeding). Specifically, the insect damage types (DTs; Labandeira et al., 2007) include hole and surface feeding (DTs 1–5, 29, 50; Figure 4E), margin feeding (DTs 12–15; Figure 5A), possible galling (?DT11), and possible mining (?DT41).

*Notes on specimen selection—Caffapanax canessae* is circumscribed along a continuum of variation from smaller, three‐lobed blades (Figures 4E, 5) to expanded, compound‐lobed forms (Figures 1, 2, 3, 4A, B), which are the largest angiosperm leaves in the Laguna del Hunco assemblage with a maximum reconstructed leaf area >30,000 mm^2^ (Merkhofer et al., 2015). Designating specimens to represent the new species was challenging because of specimen variability and fragmentation of several specimens, as well as the presence of at least 14 other palmately‐lobed angiosperm morphotypes in the assemblage (author's observations). Thus, the holotype and two paratypes (Figures 1, 2, 3, 4A) were chosen to represent the distinctive combination of exceptionally large, palmately compound‐lobed blades with abruptly expanded medial lobes and small, narrow teeth. Additional specimens were selected conservatively to include three‐lobed and often‐fragmented five‐lobed forms (Figure 4B–D) that best exhibited the characteristic venation and diminutive teeth seen in the types.

***Genus*** **—** *Davidsaralia* Wilf gen. nov.

***Generic diagnosis*** **—**As for the type species, due to monotypy.

**Figure 7:**
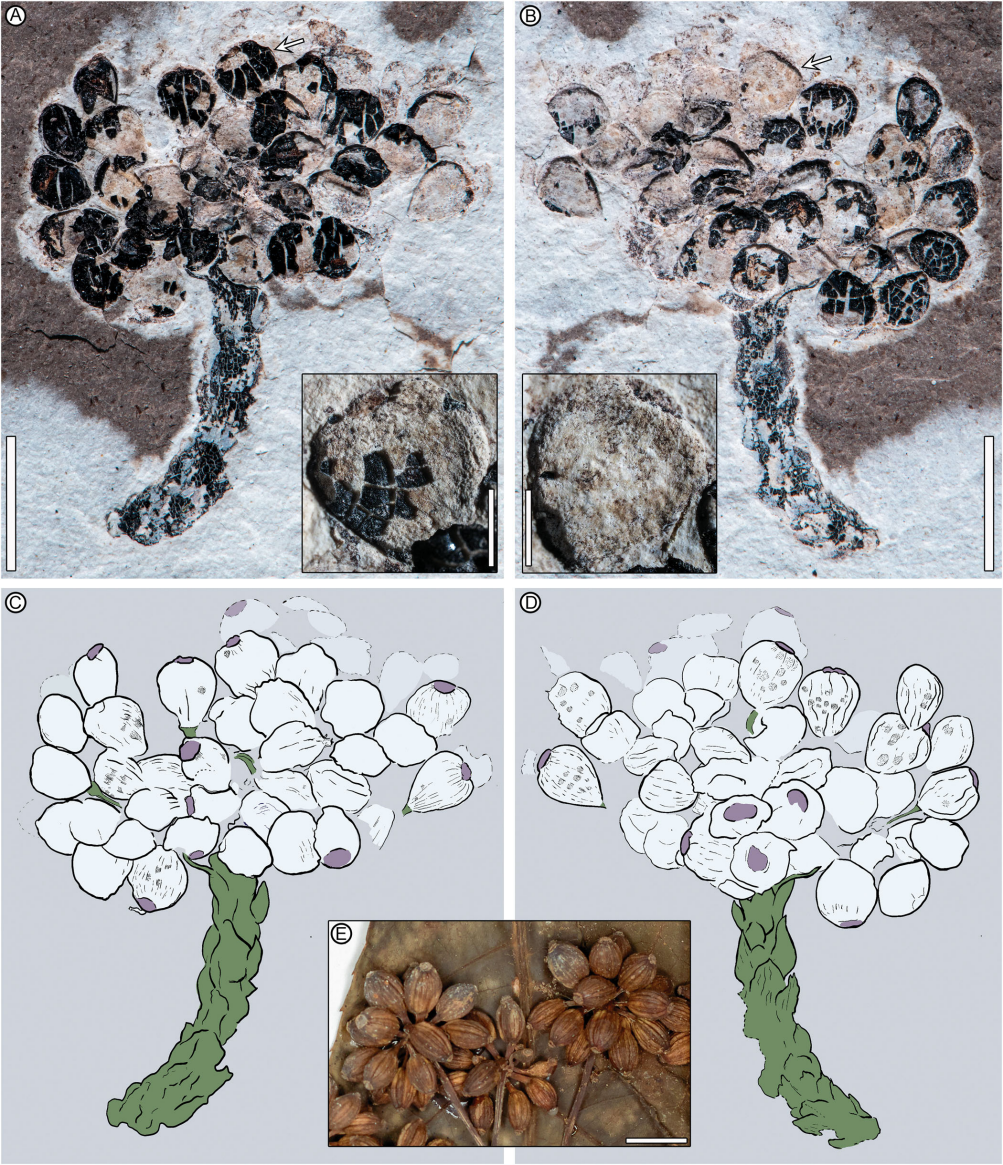
*Davidsaralia christophae* gen. et sp. nov. holotype and only specimen (A–D) and comparable infructescences of *Osmoxylon novoguineense* (E). (A, B) MPEF‐Pb 10180a (A, A inset) and counterpart MPEF‐Pb‐10180b (B, B inset). Arrows: positions of the respective fruits in the insets, whose appearances vary slightly among photos due to removal of some coal flakes. The fruits in the insets preserve remnant elongate epidermal cells, longitudinal striations, and rounded apical nectaries. (C, D) Respective overlay drawings of (A) and (B), emphasizing spiral bracts on the ultimate axis (green), preserved fruit outlines (black), pedicels (green), longitudinal fruit striations with subparallel darkened patches (stippled), and rounded apical nectary discs (pink). Drawings: Rebecca Horwitt. (E) Fertile umbellules of *Osmoxylon novoguineense* (Madang Province, Papua New Guinea, A 00934645; see Figure 8I caption for full atrribution of the same specimen), showing characteristic thickened apical nectaries, lack of styles, and longitudinal ribs delineating the carpels. Scale bars: 5 mm (A, B main panels), 1 mm (A, B insets), 1 cm (E).

***Type species*** **—** *Davidsaralia christophae* Wilf sp. nov. (Figure 7A–D).

***Holotype*** **—**MPEF‐Pb 10180 (Figure 7A–D), Laguna del Hunco, Tufolitas Laguna del Hunco of the Huitrera Formation, early Eocene (Ypresian). Collected at quarry LH27 (location given by Hajek et al., 2025), 17 March 2019. Repository: Museo Paleontológico Egidio Feruglio Paleobotany Collection (MPEF‐Pb), Trelew, Chubut, Argentina.

***Etymology*** **—**The new generic and specific epithets honor two legendary explorers and supporters of botanical research worldwide, Christopher Davidson (1944–2022) and Sharon Christoph of Flora of the World (https://floraoftheworld.org; Taylor et al., 2023). *Davidsaralia christophae* is, to my knowledge, the first fossil among at least 11 taxa named for Dr. Davidson (Taylor et al., 2023). Dr. Davidson had lifelong interests in geology and paleobotany, and he participated vigorously in the 2019 field trip to Laguna del Hunco, where he unearthed the holotype and only specimen of the new taxon (Figure 7A–D).

***Diagnosis*** **—**Umbellate infructescence with more than 40 elliptical to obovate fruits borne in a dome‐shaped cluster on slender pedicels emerging from the apex of an ultimate axis segment with ovate, adpressed, spirally deployed bracts over its full preserved length. Fruits with a rounded, thickened, flattened epigynous disc.

***Specific description*** **—**The specimen (Figure 7A–D) is an isolated umbellule most likely derived from a larger compound umbellate infructescence, composed of the distal portion of an ultimate axis subtending a dome‐shaped (hemispheric) cluster, height 11 mm, width 18 mm, of numerous pedicellate, solitary, drupe‐like fruits emerging from the axis apex. The ultimate axis portion has preserved length 10.3 mm, width 2.3 mm and is fully clothed in spirally deployed ovate, adpressed bracts with acute apices; distal bracts subtend the infructescence; bract length to 1.7 mm, width to 0.6 mm. Pedicels are slender, width 0.2 mm, expanded distally to 0.5 mm. Fruits number ca. 40 visible; more are presumed to be flattened underneath the exposed compression surfaces. The cleavage plane confined several fruits to appearing only on the part or counterpart. Fruits are elliptical to obovate in shape, length to 2.8 mm, width to 2.4 mm, presumed fleshy outer layers coalified, ovary inferior, with numerous fine longitudinal striations present. Fragmentary darkened patches occur subparallel to the striations, potentially indicating up to nine (18 by symmetry) partly coalified zones of thickened or protruding rib tissue. Epidermal cells are narrow and linear. Calyx rim not observed. Epigynous disc (interpreted as a nectary or stylopodium) well marked at the fruit apex, circular, thickened, flattened to depressed, diameter 0.7–1.5 mm. Styles (stylodia) apparently absent; seeds not observed (some objects near the fruit apices appear to be extruded plant matter or debris).

## DISCUSSION

### *Caffapanax*: comparisons outside Araliaceae

The leaf architecture of *Caffapanax canessae* gen. et sp. nov. differs from superficially similar non‐araliads (taxa mentioned here are not illustrated to conserve space but are viewable using online resources, see Methods). *Cochlospermum* (Bixaceae, e.g., *C. vitifolium*) species can have large, palmately compound, toothed leaves, sometimes with enlarged medial lobes. However, the leaves do not develop compound lobes, and when toothed, their secondary and exterior tertiary veins typically terminate in the tooth sinus (Carvalho et al., 2011) versus the tooth apex in the new fossils (see also “Other Patagonian fossils and the *Cochlospermum* problem”).

Some Passifloraceae (e.g., *Passiflora vitifolia*) have five‐lobed leaves with small sublobes. In addition to their much smaller blades and sublobes, these leaves have glandular teeth and markedly different base architecture from the fossils, including lobate bases with naked lateral, palinactinodromous primary veins. Among the palmately lobed Euphorbiaceae (Carvalho et al., 2011), *Jatropha* species (e.g., *J. macrorhiza*) can have compound lobes, but the primary lobes are subequal (without an abruptly larger medial lobe), and the overall leaf architecture, including spinose teeth and lack of agrophic veins, is entirely different from the fossils. *Cnidoscolus* species (e.g., *C. tubulosus*) lack compound lobes, and their teeth are sparse. *Melanolepis* species (e.g., *M. multiglandulosa*) may be compound lobed; however, the primary lobes are subequal, the lobe and tooth sinuses are broadly rounded, and the tertiary fabric is strongly opposite percurrent.

Other woody taxa with palmately lobed species can be excluded. Compound lobes occur in species of *Liquidambar* (Altingiaceae; e.g., *L. orientalis*), *Ficus* (Moraceae; e.g., *F. carica*), Malvaceae (*Hibiscus* spp.), Sapindaceae (*Acer* spp.; e.g., *A. tenuifolium*), Platanaceae (*Platanus* spp.), and Grossulariaceae (e.g., *Ribes watsonianum*), but in each case the primary lobes are subequal, and any sublobes are comparatively smaller and more numerous than in the fossils. Vitaceae leaves sometimes have five or more lobes with small sublobes (e.g., *Vitis arizonica*) but typically have subequal lobes, angular lobe sinuses, lobate bases, and weaker basal‐primary organization than the fossils. *Stigmaphyllon urenifolium* (Malpighiaceae) leaves can have a pinnately sublobed medial lobe evocative of the fossils, but they also have untoothed margins and lobate bases with naked basal veins. Many additional taxa with palmate lobation have non‐compound or subequal lobes, including in Caricaceae (*Carica*, *Jarilla*) and Malvaceae (*Cola*, *Triplochiton*). A few Proteaceae have complex lobing but do not resemble the fossils, including species of *Petrophile*, *Stenocarpus*, and *Knightia*.

Herbaceous, non‐aquatic angiosperms rarely fossilize due to their low biomass and indehiscent leaves (e.g., Behrensmeyer et al., 2000), and there are no definite examples of this type of leaf fossil from Laguna del Hunco. Several families have herbaceous species with palmately compound‐lobed, toothed leaves that sometimes have an enlarged medial lobe, including Apiaceae (e.g., *Astrantia*, *Eryngium*, *Heracleum*), Berberidaceae (*Podophyllum*), Cucurbitaceae (*Cucurbita*, *Cyclanthera*), Geraniaceae (*Geranium*), Gunneraceae (*Gunnera*; formerly in Araliaceae: Lindley, 1846), and Dioscoreaceae (*Tacca*). However, in each case, the gross morphology and venation differ substantially from the fossils. For example, *Gunnera tinctoria*, which occurs widely in Patagonia, has famously large, palmately lobed leaves but differs in its lobate bases, naked basal veins, palinactinodromous primary venation that characteristically bifurcates, and glandular teeth in multiple orders (Fuller and Hickey, 2005).

### *Caffapanax* **: comparisons in Araliaceae**

**Figure 8:**
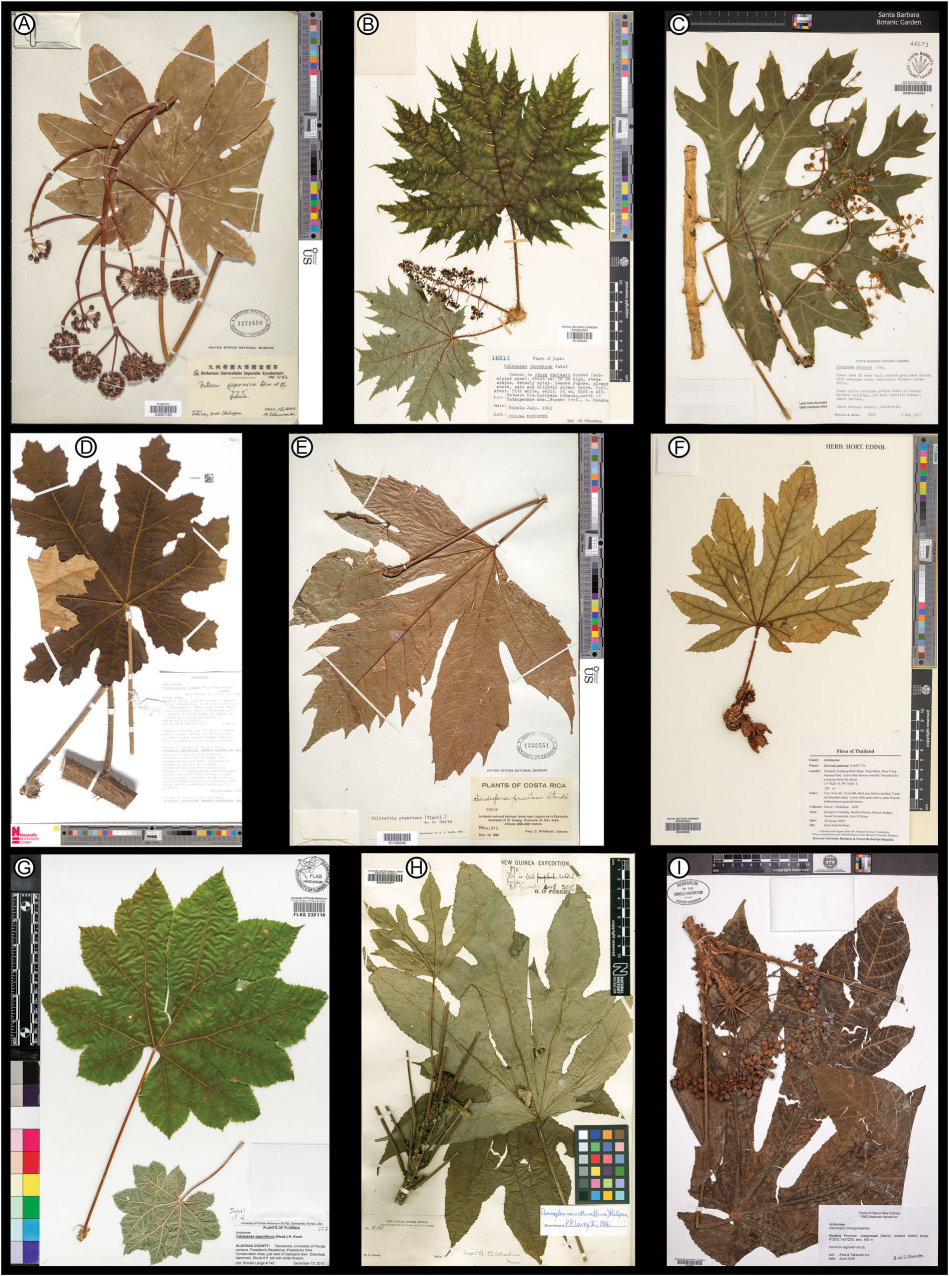
Herbarium sheets (downloaded images) of selected palmately compound‐lobed Araliaceae species for comparison with *Caffapanax canessae* gen. et sp. nov. in Figures 1, 2, 3, 4, 5, 6. (A) *Fatsia japonica* (Chikuzen, Japan), US 03067185, http://n2t.net/ark:/65665/3a334c50e-dd91-4d01-8a3e-958df72281e9. (B) *Oplopanax horridus* (Nagano Prefecture, Japan), E 01255406, https://data.rbge.org.uk/herb/E01255406. (C) *Oreopanax peltatus* (cultivated, Santa Barbara, California), SBBG 244095, via GBIF, https://cch2.org/imglib/cch2/SBBG/SBBG244/SBBG244095.jpg. (D) *Harmsiopanax ingens* (central Irian Jaya, Indonesian New Guinea), L 3730124, https://data.biodiversitydata.nl/naturalis/specimen/L.3730124. (E) *Dendropanax praestans* (San José, Costa Rica), US 01156342, http://n2t.net/ark:/65665/37fefdb13-7f17-4212-9764-8a01d59fba68. (F) *Trevesia palmata* (Huay Yang National Park, Thailand), E 00396382, https://data.rbge.org.uk/herb/E00396382. (G) *Tetrapanax papyrifer* (cultivated, Gainesville, Florida), FLAS 232116, https://specifyportal.floridamuseum.ufl.edu/herbarium. (H) *Osmoxylon micranthum* (Sogeri region, Papua New Guinea), BM 000944877, https://data.nhm.ac.uk/object/db81365c-6544-43a9-baed-8c9daecaf864. (I) *Osmoxylon novoguineense* (Madang Province, Papua New Guinea; see also Figure 7E) A 00934645, https://kiki.huh.harvard.edu/databases/specimen_search.php?barcode=00934645. License status: A, D, E, H: CC0 1.0; B, F: CC BY 4.0; C, G: CC BY‐NC 4.0; I: with permission of The Herbarium of the Arnold Arboretum of Harvard University.

**Figure 9:**
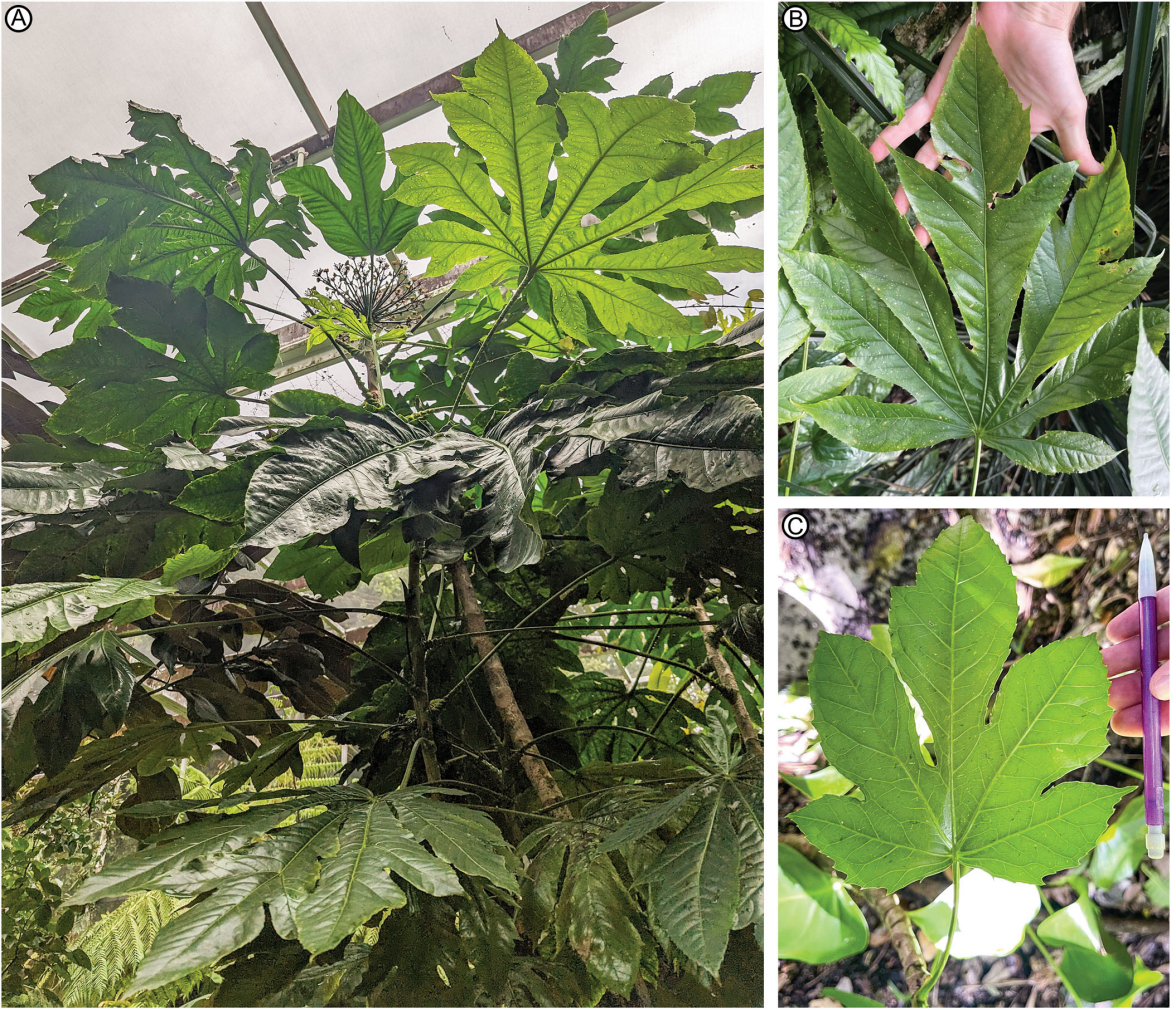
*Osmoxylon novoguineense* in living collections of the Fairchild Tropical Botanical Garden, Miami Florida. (A) Small flowering tree (specimen RPH‐B3, 2013−0491) showing wide foliar variation in size, number of lobes (3–9), medial lobe expansion, and development of sublobes, thus encompassing most of the variation seen in the fossils (Figures 1, 2, 3, 4, 5, 6) on one plant. Note three‐lobed leaf at top (cf. Figures 5A) and 5–7‐lobed blades with expanded medial lobes at bottom right; cf. Figures 1, 2, 3) that especially resemble the fossils. (B) Leaf from tree in A with seven lobes (five of them with sublobes) and expanded medial lobe. (C) Juvenile leaf with five lobes and incipient sublobes (Plot 151, 2021‐0063). Pencil length = 15 cm.

Many araliaceous species have large, palmately compound‐lobed, cordate, toothed leaves similar to those of *Caffapanax canessae* (Frodin, 1973; Philipson, 1979). These taxa often display dramatic variation in leaf size, shape, and organization, even within individuals. The upper size ranges easily exceed standard herbarium sheets and thus have limited representation in collections (Philipson, 1970, 1979; Plunkett et al., 2004), and many species from remote locations are undersampled. Although general leaf features of Araliaceae are well described, no work provides the detailed level of architectural descriptions needed for adequate fossil comparisons or phylogenetic analysis. Thus, the comparisons made here are primarily based on my surveys of herbarium material and the literature (see Materials and Methods), targeting genera that have diagnostic features of the fossils within their often‐wide ranges of variation, which make a full tabulation by genus impractical. Exemplars for the following discussion (Figures 8, 9) were selected to maximize similarities to the fossils within their genera.

Plunkett et al. (2018) noted palmate lobation in 18 of the 46 Araliaceae genera: *Brassaiopsis*, *Cussonia*, *Dendropanax*, *Fatsia*, *Harmsiopanax*, *Hedera*, *Hydrocotyle*, *Kalopanax*, *Macropanax*, *Merrilliopanax*, *Metapanax*, *Oplopanax*, *Oreopanax*, *Osmoxylon*, *Seemannaralia*, *Sinopanax*, *Tetrapanax*, and *Trevesia*. Most of these can be eliminated easily from having close affinities with the new fossils, such as *Hydrocotyle*, an herbaceous aquatic‐semiaquatic genus whose ca. 180 species are entirely unlike the fossils; *Fatsia*, which lacks expanded medial lobes, although some leaves have pinnate sublobes (Figure 8A); and *Sinopanax*, which has shallow lobes unlike the fossils. Of the remainder, pinnate sublobes, as seen in the fossils, are common in *Harmsiopanax*, *Oplopanax*, *Oreopanax*, *Osmoxylon*, *Tetrapanax*, and *Trevesia* and rare in *Cussonia* and *Dendropanax*.

Three of these eight genera that exhibit pinnate sublobes are otherwise clearly different from *Caffapanax*. *Cussonia* (tropical to southern Africa and adjacent islands; 20 species) has highly variable foliage that is rarely palmately lobed, and these examples (e.g., *C. arborea*, *C. spicata*) are not similar to the fossils. Their lobe incision is characteristically at a low angle to the primary vein and approaches or reaches the midvein, splitting the lobe into segments, and there is no expanded medial lobe. *Oplopanax* (Figure 8B; northeastern Asia and northern North America; three species) leaves have a lower aspect ratio, a frequently spiny surface, and usually more convex, less incised lobes than the fossils. The medial lobe is not expanded, and the sublobes are small, numerous, triangular, and further divided, unlike the few, large sublobes of the fossils. The teeth are markedly different from the fossils in their sharply triangular shape, close spacing, and continuous size variation grading into the sublobes. *Oreopanax* (tropical Americas; 147 species) has diverse, often peltate foliage types and compound‐lobed forms in several species (*O. ecuadoriensis*, *O. incisus*, *O. peltatus*; Figure 8C). The lobes are mostly narrower than in the fossils, the medial lobe is not expanded, and the teeth are much larger than those of the fossils when present.

The five remaining genera (Figures 8D–I, 9), all with distributions including Malesia or South China, exhibit clearer similarities to the *Caffapanax* fossils in their range of variation (Miquel, 1863; Boerlage, 1887; Harms, 1921; Liao, 1965; Frodin, 1973; Stone, 1978; Lowry, 1989; Philipson, 1979, 1995; Conn and Damas, 2019; Utteridge and Frodin, 2021). None has a reliable fossil record. *Harmsiopanax* (Malesia; three species) generally has large, multilobed, frequently compound‐lobed, toothed leaves like the fossils, although some leaves are smaller with fewer lobes (Philipson, 1973). Unlike the fossils, the teeth are usually compound, triangular, and closely spaced, the blade and petiole surfaces are conspicuously spiny, and the medial lobe is not abruptly expanded. The species that most resembles the fossils is *H. ingens* (Figure 8D; New Guinea). *Dendropanax* (Malesia, East Asia, tropical and subtropical Americas; 95 species) usually has undivided to 2–5 palmately lobed, untoothed leaves; the teeth are sparse when present. Compound lobing is rare, but forms comparable to the fossils occur in *D. praestans* (Figure 8E; Central America), which can develop an expanded medial lobe and deep lobe incisions. Unlike the fossils, *D. praestans* has large teeth that grade in size into the sublobes, which are much less incised than in the fossils.

*Trevesia* (South China to Malesia; eight species) is well known horticulturally for specimens with attractive webbed‐digitate, palmately lobed (but appearing compound) foliage (Jebb, 1998; Wen et al., 2007). Some species (*T. palmata*, Figure 8F; *T. sundaica*) can have palmately compound‐lobed, toothed leaves. These examples differ from *Caffapanax* in that they lack an abruptly expanded medial lobe and have many more lobes (nine or more vs. 3–5). *Tetrapanax papyrifer* (Figure 8G; China and Taiwan, the sole species of its genus) leaves may be toothed or untoothed and differ from the fossils in usually having many more primary lobes (7–11 vs. 3–5), a strongly lobate base (vs. cordate in the fossils), and bifurcating primaries that often terminate in bifid lobe apices (Perdue and Kraebel, 1961; Liao, 1965). However, some juvenile foliage is relatively small and 5‐lobed.

**Figure 10:**
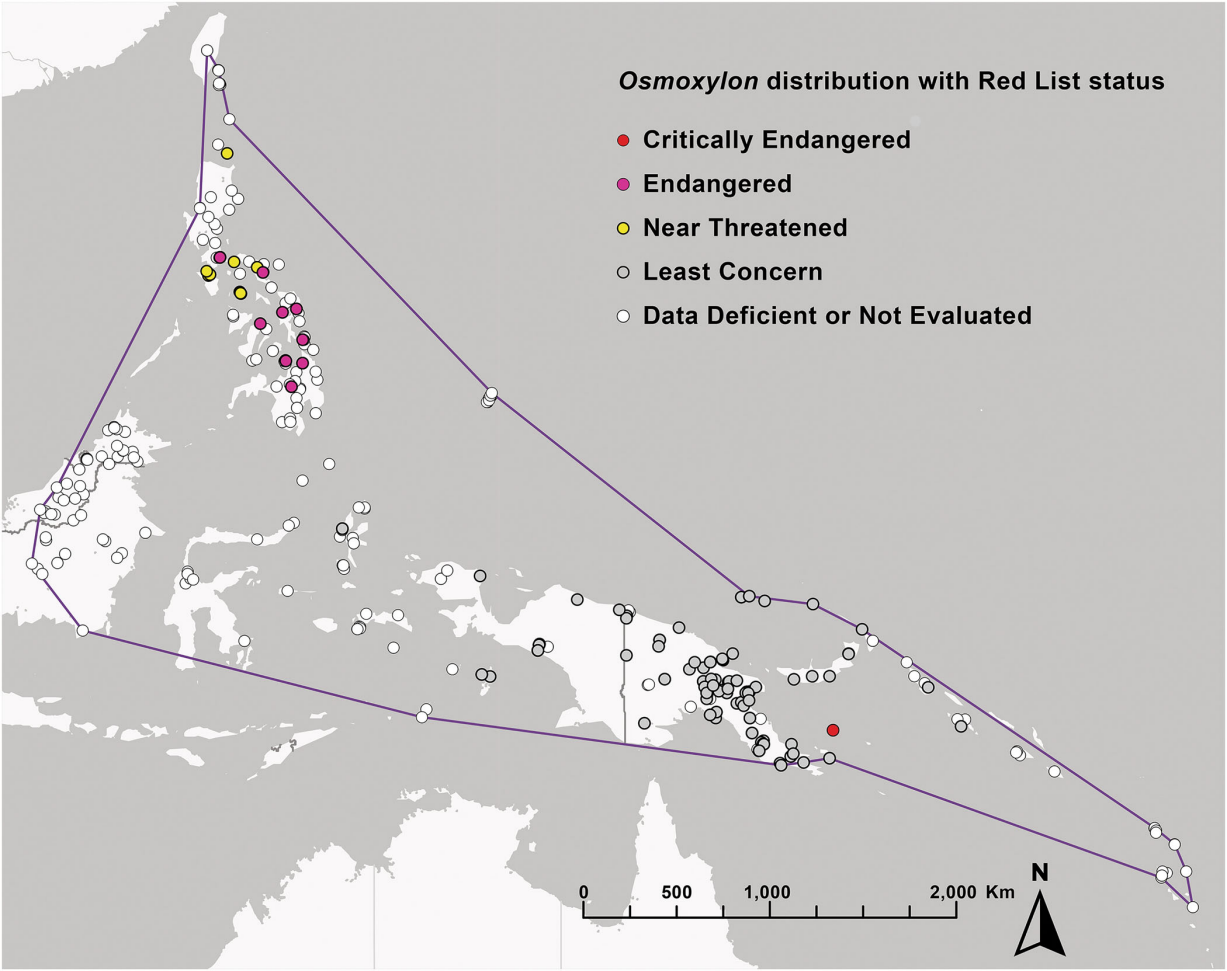
Distribution of *Osmoxylon*, coded by the IUCN Red List status (IUCN, 2024) of the species vouchered at each location. Data downloaded from GBIF (www.gbif.org), including Red List status, and pre‐filtered to include only preserved specimens (herbarium vouchers) with coordinates, from countries in the known range of the genus (the data can be re‐queried by species and other fields by retrieving the download at https://doi.org/10.15468/dl.f553ec). Cultivated specimens were removed manually. The genus range also includes the Mariana Islands, not shown here because the GBIF data for that location did not include herbarium vouchers. Map constructed in ArcGis Pro 3.4.0 (ESRI, Redlands, CA, USA).

*Osmoxylon* (Figures 7E, 8H, 8I, 9, 10; 61 species) consists mostly of understory trees and ranges from Wallacea to Taiwan, Vanuatu, and the Mariana Islands; the genus reaches its highest diversity in eastern Malesia, especially the Philippines. There are numerous taxonomic treatments of *Osmoxylon* (Miquel, 1863; Boerlage, 1887; Harms, 1921; Liao, 1965; Stone, 1962, 1978, 1983; Lowry, 1989; Conn and Frodin, 1995; Philipson, 1976, 1979, 1995; Frodin, 1973, 1998; Costion and Plunkett, 2016; Conn and Damas, 2019; Utteridge and Frodin, 2021). Frodin (1998) reviewed its nomenclatural history, and Philipson (1976) listed many of the vernacular names. Of 27 *Osmoxylon* species assessed for extinction risk in the IUCN Red List (excluding Data Deficient entries), 19 (70%) are listed as vulnerable (two species), three as near threatened, 10 as endangered, and four as critically endangered (Figure 10; IUCN, 2024; 26 of 27 assessments are from the past 5 years). The top threats are forest loss from agriculture, logging, and urbanization.

*Osmoxylon* foliage characteristically varies, even on individual plants (Figure 9), in lobe number and in form, from simple unlobed to palmately compound, palmately lobed, and palmately compound‐lobed (Harms, 1921; Stone, 1962; Conn and Frodin, 1995; Philipson, 1979, 1995). Because many rare species are underrepresented in collections, a comprehensive evaluation of leaf architecture is currently not possible for this genus. Nevertheless, there are several *Osmoxylon* species with foliage comparable to the *Caffapanax* fossils in having combinations of a variable number of lobes, some of them compound with few sublobes, often‐expanded medial lobes, comparably small, narrow, nonglandular teeth, and fimbrial veins or intramarginal veins running close to the margin (examples: *O. luzoniense*, *O. micranthum*, *O. novoguineense*, *O. pulcherrimum*, *O. sessiliflorum*).

*Osmoxylon micranthum* (Figure 8H; New Guinea, 700–2400 m a.s.l.) has a similar lobe count (usually 3–5), enlarged medial lobe, lobe divisions, and general aspect to the fossils; minor differences are the deeper lobe incisions and frequent small sublobes not seen in the fossils. *Osmoxylon novoguineense* (New Guinea, Solomon Islands, 0–1600 m a.s.l.; Figures 8, 9) is comparable to *Caffapanax* in overall architecture, and its three‐lobed leaf forms, usually located distally near the inflorescences (Figure 8A; Philipson, 1979, 1995), closely resemble some of the smaller leaf fossils (Figure 5A). The differences include that most leaves have more lobes than the fossils (7–9), the lobes are narrower and more incised, and the medial lobe expansion is inconsistent.

Based on these comparisons, *Caffapanax* can be placed confidently in Araliaceae, although not in a living genus. The fossils' leaf architecture closely resembles some *Osmoxylon* species, but in addition to the minor differences in leaf architecture, that genus has conspicuous diagnostic features not yet found in the fossils, including a collared petiole base in most species (the base of the petiole was not recovered in the fossils: see Figure 4D) and compound umbellate inflorescences with three primary branches, the middle branch composed of sterile, berry‐like (bacciform) flowers (pseudofruits). Although *Davidsaralia* (Figure 7A–D) is a fertile umbellule that may be analogous to a lateral, fertile branch of these structures, its relationships with *Caffapanax* and living genera cannot yet be confirmed (see *Davidsaralia* comparisons, below). Therefore, a new generic name for the leaf fossils is appropriate. Notably, all five of the similar extant genera (Figures 8D–I, 9) are distributed in Malesia to South China and Oceania, although most species of one genus (*Dendropanax*) are neotropical (Philipson, 1979; Qibai and Lowry, 2007; Li and Wen, 2016; Coca‐de‐la‐Iglesia et al., 2022). These extant ranges are consistent with the predominant Indo‐Pacific biogeographic signal in the overall flora (see introduction).

### **Other Patagonian leaf fossils and the** *Cochlospermum* **problem**

A small set of Araliaceae‐like Patagonian leaf fossils suggests a more extended history of the family in the region. The examples below are illustrated elsewhere, as cited. None compares closely with the new *Caffapanax* leaf fossils.

The early Paleocene Salamanca Formation flora from southern Chubut contains several palmately lobed leaf species (Berry, 1937; Iglesias et al., 2007, 2021). The most abundant, “*Cissites*” *patagonica* (e.g., Iglesias et al., 2021: Figures 10, 11), is superficially similar to some smaller forms of *Caffapanax* (Figure 5B, C). However, the “*Cissites*” leaves are unlobed to three‐lobed, with noncordate bases, densely serrate margins, and two orders of small, glandular teeth (*Caffapanax* is cordate, with one order of more widely spaced, nonglandular teeth). A more likely candidate for Araliaceae from this flora is morphotype SA019 (Iglesias et al., 2021: figures 15, 17), which has an untoothed blade bearing up to six primary lobes and small, pinnate sublobes. Comparable morphologies occur in some *Oreopanax* species (e.g., *O. dactylifolius*, *O. geminatus*).

From the earliest middle Eocene (ca. 47.8 Ma) Río Pichileufú flora from Río Negro Province in Argentine Patagonia (Berry, 1935, 1938; Rossetto‐Harris and Wilf, 2024), several long‐petiolate, trilobed, toothed leaves were assigned to *Oreopanax guinazui* (Berry, 1938). These specimens are variable in architecture and probably encompass several biological species with unknown family relationships. From the same assemblage, Rossetto‐Harris and Wilf (2024: Figure 4F) reported a fragmentary large, lobed, toothed leaf assigned to their morphotype GZ018. The specimen lacks a base, and it is not possible to distinguish whether it is pinnately lobed or the apical portion of a palmately compound‐lobed leaf like *Caffapanax* (cf. Figure 4B). The GZ018 specimen may belong to Proteaceae (Rossetto‐Harris and Wilf, 2024), but a specimen with a preserved base is needed to resolve this issue.

The Río Pichileufú flora also has palmately lobed, long‐petiolate leaves of “*Cochlospermum*” *previtifolium* Berry (1935, 1938; see also section Comparisons outside Araliaceae). Berry (1935, 1938) considered Araliaceae as an alternative family for this species, but there is no compelling evidence for this idea, and the assignment to *Cochlospermum* is also unjustified. Like the living genus (and unlike *Caffapanax*), these fossil leaves have subequal lobes with no sublobes, but they lack the distinctive generic feature of prominent veins running to the tooth sinuses (see Carvalho et al., 2011). The isolated fruit fossil assigned to the same name (Berry, 1935, 1938; Rossetto‐Harris and Wilf, 2024: figure 14D) also shows no indication of typical *Cochlospermum* features (Jennings, 2021) and requires further study. At Laguna del Hunco, the possible presence of *Cochlospermum* (Wilf et al., 2005a; González, 2008; Merkhofer et al., 2015) is currently supported only by a faintly preserved five‐lobed leaf with a long petiole (MPEF‐Pb 7974), which lacks key venation and tooth details, and one fruit fragment (MPEF‐Pb 1602). On review here, these fossils are not consistent with *Caffapanax*, Araliaceae, “*Cochlospermum*” *previtifolium*, or living *Cochlospermum*.

### *Davidsaralia* **comparisons**

Few plant families have species with the combination of umbellate infructescences and solitary fruits with inferior ovaries observed in *Davidsaralia christophae* gen. et sp. nov. (Figure 7A–D), including Alstroemeriaceae, Apiaceae, Araliaceae, Cornaceae, and Lauraceae (e.g., Watson and Dallwitz, 1992 onward). Of these, only Apiaceae and Araliaceae have species with infructescences plausibly resembling the fossil, including the presence of an often‐dense, dome‐like cluster of fruits with prominent epigynous discs, which are nectaries formed from the ovary roof (stylopodia; Erbar and Leins, 2010). However, most Apiaceae fruits are schizocarps, whereas the fossil fruits are undivided, fleshy, and drupe‐like in appearance, as in many Araliaceae taxa (Philipson, 1979; Plunkett et al., 2018). Additionally, Apiaceae are two‐carpeled, with two styles (stylodia; Erbar and Leins, 2010) emerging from a relatively narrow, bipartite stylopodium, whereas Araliaceae usually have 2–5 carpels but may have up to 100 or more per fruit. In Araliaceae, styles may be present or absent; when absent, the stigmas may be pustulate and flush with the stylopodium, as in *Osmoxylon* (Figure 7E). In *Davidsaralia*, the discs are wide and undivided, and no styles are visible, aligning the fossil with Araliaceae (Wen et al., 2001; Erbar and Leins, 2010; Plunkett et al., 2018). In living Araliaceae, the fruit ribs (e.g., Figure 7E) usually represent the external pyrene surfaces and are thus equal in number to the carpels, as seen in *Osmoxylon* (Philipson, 1979). Although inconclusive due to preservation, the longitudinal striations on the fossil fruits and subparallel coalified patches (Figure 7A–D) may represent the remnants of thickened or protruding fruit ribs (Figure 7E); if so, the number of ribs (and thus carpels) per fruit would be at least 18 by symmetry, far greater than the two carpels of Apiaceae.

Following the hypothesis that *Davidsaralia* had 18 or more carpels, comparable araliad genera include *Plerandra* (up to 19 carpels), *Heptapleurum* (range unknown), *Polyscias* (up to 16–24), and *Osmoxylon* (1–25 + ; Lowry et al., 2013; Plunkett et al., 2018). *Plerandra* has markedly fewer fruits per umbellule than the fossil; *Heptapleurum* (formerly under *Schefflera*) comprises several hundred species, many of them undescribed, whose reproductive characters have not yet been tabulated (Lowry and Plunkett, 2020). *Polyscias* species have diverse infructescence types and fruit morphologies, some of which may overlap with the fossil, but the styles are often prominent (Philipson, 1979; Lowry and Plunkett, 2010; Plunkett et al., 2018). *Davidsaralia* is more similar to *Osmoxylon*, which has numerous drupaceous fruits and no visible styles; the stigmas may be reduced to pustulate structures on the disc (Figure 7E; Philipson, 1979; Conn and Frodin, 1995; Lowry and Plunkett, 2024) that are unlikely to be preserved in these fossils. Thus, *Osmoxylon* appears to be the closest known genus to *Davidsaralia* based on current evidence, but the fossil is too incomplete for a comprehensive comparison across Araliaceae.

Extant species of *Osmoxylon* are distinctive for their compound umbellate inflorescences comprising three primary branches: a middle branch with sterile, berry‐shaped (bacciform) flowers (pseudofruits), and two fertile lateral branches (Philipson, 1976; Utteridge and Frodin, 2021). Based on the presence of well‐developed nectaries and possible fruit ribs, *Davidsaralia* was most likely fertile; thus, it would be analogous to an *Osmoxylon* lateral fruit umbellule.

To my knowledge, there are no living araliad species with spirally arranged bracts that fully envelop the ultimate inflorescence axis, as in *Davidsaralia*. Usually, only a pair (or a few pairs) of opposite or subopposite bracts is found along the axis (Philipson, 1979). This feature, in addition to the secondary considerations of age and location, supports the recognition of a new genus. The similarities of both the *Caffapanax* leaves and the *Davidsaralia* umbellule to *Osmoxylon* suggest that both fossilized organs came from the same *Osmoxylon*‐like source species. The absence of evidence for other araliaceous species in the well‐sampled, diverse Laguna del Hunco flora also supports this idea, but the lack of organic attachments and the fragmentary nature of the single *Davidsaralia* specimen make separate names necessary.

### Araliaceae fossil record and biogeography

Early records of araliaceous pollen come from the Late Cretaceous (early Campanian) of Wyoming and the Paleocene and Eocene of Europe, Greenland, and western North America, including *Aralia* pollen from the early‐middle Eocene Princeton Chert, British Columbia (Manchester et al., 2015; Geier et al., 2024). The macrofossil record of the family is surprisingly scarce (Frodin and Govaerts, 2003; Martínez‐Millán, 2010; Mitchell et al., 2012; Plunkett et al., 2018; Pan et al., 2025). Manchester et al. (2015) considered the only reliable occurrences to be *Paleopanax oregonensis* fruits from the middle Eocene Clarno Nut Beds of Oregon, similar to extant *Metapanax* (*Pseudopanax*) *davidii* (Manchester, 1994), and fruits from the middle Eocene Puryear clay pit of Tennessee, USA, later named as *Paleopanax puryearensis* (Na et al., 2020). Subsequent reports are all Neogene (and thus post‐Gondwanan), including early Miocene *Astropanax* foliage and pollen from the Mush Valley flora of central Ethiopia (Pan et al., 2025), middle Miocene *Pseudopanax* sp. flowers with in situ pollen and potential *Pseudopanax* and *Schefflera* foliage from the Hindon Maar deposit in southern New Zealand (Kaulfuss et al., 2018, 2022), and Pliocene *Aralia stratosa* endocarps from Yunnan, China (Zhu et al., 2015).

Putative Araliaceae leaves appear throughout the paleobotanical literature and are especially common in pioneer‐era monographs, which included dozens of species of “*Aralia*” and other taxa (e.g., Knowlton, 1919). Some of these fossils could be correctly assigned to the family, but none has been validated using modern methods, and several have been rejected (e.g., Manchester et al., 2015, 2024). Among these, *Dendropanax eocenensis* leaves with cuticles from the middle Eocene Claiborne Group in Tennessee (Dilcher and Dolph, 1970) were considered among the oldest reliable fossils of the family (Martínez‐Millán, 2010; Nicolas and Plunkett, 2014); however, Manchester et al. (2015) disputed the genus and family identifications based on inconsistent leaf architecture and cuticle characters.

*Parafatsia subpeltata*, comprising multilobed leaves from the middle Eocene Maslin Bay flora of South Australia, was thought to be a reliable Gondwanan record of Araliaceae (Blackburn, 1981). However, Carpenter et al. (2006) re‐assigned this species to the Proteaceae based on cuticular characters, and the macromorphology is not close to *Caffapanax* (nor to any living Proteaceae), including its more numerous (11), subequal, simple lobes. Later, Pole (2019) reported a 5–6‐lobed morphotype similar to *P. subpeltata* (his parataxon DINM‐JJD) from the early Eocene of Dinmore, Queensland. From the post‐Gondwanan southern hemisphere, in addition to the Hindon Maar fossils (Kaulfuss et al., 2018, 2022), Oligocene/Miocene foliage occurrences are listed as aff. *Polyscias* and aff. Araliaceae from the Latrobe Coal Measures in southeastern Australia (Blackburn and Sluiter, 1994) and cf. *Schefflera* from the Gore Lignite Measures of southern New Zealand (Ferguson et al., 2010). A few palynological records of Araliaceae have come from various post‐Gondwanan sites in Australia and New Zealand (compiled in Hill, 1994; Kooyman et al., 2014).

The new Araliaceae fossils from early Eocene Patagonia update the biogeographic context of the family by placing its oldest macrofossil occurrences in Gondwana. Unsurprisingly, in light of its prior fossil record, biogeographic studies of Araliaceae have mainly focused on movements and disjunctions in the northern hemisphere, especially involving Asia and North America (Wen, 1998, 2000; Wen et al., 1998, 2001; Valcárcel et al., 2014; Li and Wen, 2016; Zuo et al., 2017; Valcárcel and Wen, 2019; Coca‐de‐la‐Iglesia et al., 2022; Kang et al., 2023). Nevertheless, several authors have suggested Gondwanan history in Araliaceae (Raven and Axelrod, 1974; Mitchell et al., 2012), and Philipson (1979, p. 1) observed that “the family and its centres of distribution are largely found within the land masses derived from ancient Gondwanaland.” Nicolas and Plunkett (2014) inferred a Late Cretaceous, Australasian (thus Gondwanan) origin for the family from molecular data, although the Old World tropics have also been proposed (Wen et al., 2001).

Basal polytomies and short branch lengths in Araliaceae phylogenies have been interpreted as legacies of rapid radiations that followed Gondwanan breakup (Plunkett et al., 2004; Valcárcel et al., 2014). However, the potential relationships of *Caffapanax* and *Davidsaralia* as extinct relatives of *Osmoxylon*, which occupies a near‐basal, isolated position in most family phylogenies (Plunkett and Lowry, 2001; Lowry et al., 2004; Plunkett et al., 2004; Wen et al., 2001, 2008; Li and Wen, 2016; Kang et al., 2023), are more consistent with the idea of early divergences and extinctions before the breakup. This idea parallels the shift in biogeographic interpretations of Solanaceae resulting from the discovery at Laguna del Hunco of two species of lantern fruits from the derived genus *Physalis*. These fossils, along with subsequent discoveries, increased the evidence for early Solanaceae diversification and overland movements in Gondwana (and elsewhere), compared to the prior emphasis on post‐Gondwanan overwater dispersals out of South America (Wilf et al., 2017; Deanna et al., 2020, 2023).

Relevant molecular data for this discussion include those of Kang et al. (2023), who suggested that the ancestral area of *Osmoxylon* was Australia, which was part of Gondwana during the early Eocene, but that the generic divergence was Oligocene/Miocene (post‐Gondwanan). However, Zuo et al. (2017) reconstructed a Southeast Asian origin for *Osmoxylon*, conflicting with Kang et al. (2023) and the new fossil evidence, but they estimated a late Paleocene generic divergence that is consistent with the fossils. Thus, *Caffapanax* and *Davidsaralia* support elements of both hypotheses (Zuo et al., 2017; Kang et al., 2023), which otherwise conflict with each other. Precise phylogenetic placement of the fossils that may better resolve these issues will require additional discoveries of more complete material, but the *Caffapanax* leaves (and possibly the *Davidsaralia* fruits) are in all likelihood related to living Araliaceae of Malesia and surrounding areas. The new fossils mark a significant addition to the taxa illustrating the floristic connections of Eocene Laguna del Hunco and extant Indo‐Pacific rainforests, which share *Todea*, *Agathis*, *Dacrycarpus*, *Papuacedrus*, *Castanopsis*, *Gymnostoma*, *Macaranga*, and many more genera (see introduction).

### Paleoenvironmental and paleoecological significance

The *Caffapanax canessae* plant (potentially including *Davidsaralia christophae*) was most likely a shrub or small tree, based on its considerable maximum leaf size consistent with rainforest understory plants and its rarity potentially indicating low biomass, similar to the habits of extant *Osmoxylon* and many other large‐leaved Araliaceae (Philipson, 1979). This interpretation adds *Caffapanax* to the diverse macrofossil taxa inferred to represent the rich Laguna del Hunco understory, including arborescent (*Todea*, *Dicksonia*) and other (*Adiantum*, Gleicheniaceae) ferns, cycads (*Austrozamia*), Solanaceae (*Physalis* spp.), climbing Menispermaceae, *Macaranga*, and woody monocots (*Ripogonum*; Carvalho et al., 2013; Carpenter et al., 2014; Jud et al., 2018; Deanna et al., 2020; Wilf et al., 2016, 2017, 2023). The associated palynoflora indicated additional richness of taxa representing low‐statured vegetation, including diverse ferns, Asteraceae, Chloranthaceae, and Rubiaceae (Barreda et al., 2020). Regarding animal interactions, in addition to the recorded insect herbivory on the fossil leaves, the fruits are suggestive of bird pollination and dispersal as postulated for living *Osmoxylon* species, although primary documentation remains limited (Stone, 1962; Costion and Plunkett, 2016).

Taphonomic biases limit the preservation of large angiosperm leaves in the plant‐fossil record (e.g., Wing et al., 2009). At Laguna del Hunco, fossil leaves are consistently smaller than those of living relatives, despite excellent preservation, large samples, and quantitative reconstructions of leaf sizes using vein scaling (Merkhofer et al., 2015). *Caffapanax* is represented by only a few specimens, and it is reasonable to assume that the living source plants had even larger leaves. Worldwide, the largest leaves are strongly associated with everwet, warm conditions, where leaf size is constrained only by biomechanical and physiological limits. The expanded lamina allows for rapid morning heating to optimize photosynthesis in the shade (Wright et al., 2017). These environments are only present today on approximately 4% of the Earth's land surface (Wright et al., 2017) and are often located in threatened, biodiverse ecosystems.

Most Malesian Araliaceae are found in everwet conditions and at elevations below ca. 2400 m, including all likely living relatives of *Caffapanax* discussed here (Philipson, 1979; van Balgooy, 1998). Thus, *Caffapanax* supports the robust signal from other nearest living relatives of Laguna del Hunco species and fossilized, drought‐sensitive plant tissues for high, aseasonal precipitation and warm conditions in early Eocene Patagonia (see introduction and Materials and Methods). The hyperdiverse, everwet biome forever disappeared from southern South America soon after Laguna del Hunco time due to Antarctic separation, increased regional volcanism, and a more seasonal rainfall regime (Dunn et al., 2015; Barreda et al., 2021; Rossetto‐Harris, 2023). Northward retreat was blocked by the subtropical arid diagonal, the lack of moist montane corridors (at ca. 35 million years before the main Andean orogeny), and tropical solar insolation and floristic incumbency (Ziegler et al., 2003; Jaramillo and Cárdenas, 2013; Wilf et al., 2013).

## CONCLUSIONS

The oldest Araliaceae macrofossils, and the first to be confirmed from Gondwana or South America, are described from Laguna del Hunco, early Eocene of Chubut, Argentina, as *Caffapanax canessae* gen. et sp. nov. leaves and a *Davidsaralia christophae* gen. et sp. nov. infructescence. *Caffapanax* encompasses rare, palmately compound‐lobed leaves that are the largest known in the diverse, well‐sampled flora. The distinctive leaf architecture of *Caffapanax* allows its confident placement in Araliaceae and narrows the list of the most comparable extant taxa to five genera, of which all have ranges that include Southeast Asia. Of these, several species of *Osmoxylon*, a genus of 61 understory shrubs and small trees with a diversity center in east Malesia, have the closest resemblance to the fossils in terms of size variation, complex lobing, and other leaf architectural characteristics. *Davidsaralia* is also similar to *Osmoxylon*, and a working hypothesis is that a single extinct species related to *Osmoxylon* was the source of the new leaf and infructescence fossils.

The large *Caffapanax* leaves are typical of species found in everwet, warm, shaded environments, only found over about 4% of the Earth's land surface today, that are imperiled from deforestation and climate change. Thus, the new fossils reinforce the well‐documented floristic and paleoenvironmental links of the Laguna del Hunco flora and late‐Gondwanan Patagonia with perhumid, often endangered Indo‐Pacific rainforests, and they add to the diverse assemblage of understory ferns and angiosperms previously recognized from the site.

Gondwanan history in Araliaceae has long been suggested (see introduction) and is now confirmed directly with the new fossils, which are the only pre‐Neogene macrofossils of the family outside North America. *Caffapanax* and *Davidsaralia* provide novel information likely to enrich the understanding of their possible nearest living relative, *Osmoxylon*, a phylogenetically isolated, early‐diverging lineage of Araliaceae with numerous threatened species. These new fossils contribute to a series of ongoing discoveries from Laguna del Hunco and other fossil sites in Patagonia that have changed established ideas about the divergence times, biogeographic history, and conservation of many plant lineages (Gandolfo et al., 2011; Wilf and Escapa, 2015, 2016; Kooyman et al., 2022; Wilf et al., 2014, 2017, 2019, 2023; Wilf and Kooyman, 2023, 2025), demonstrating the increasing importance of collecting exceptionally preserved fossils from undersampled areas.

## AUTHOR CONTRIBUTIONS

**Peter Wilf**: Conceptualization; Data curation; Formal analysis; Funding acquisition; Investigation; Methodology; Project administration; Resources; Validation; Visualization; Writing—original draft; Writing—review and editing.

## CONFLICT OF INTEREST STATEMENT

The author is an Associate Editor of the *American Journal of Botany* but took no part in the peer‐review and decision‐making processes for this paper.

## Data Availability

All new fossil materials presented in this article are curated in the Museo Paleontolόgico Egidio Feruglio Paleobotany Collection, Trelew, Argentina. An image archive containing high‐resolution versions of the composed fossil plates (Figures 1, 2, 3, 4, 5, 6, 7) and individual images of the new fossils at original resolution is deposited on Figshare: https://doi.org/10.6084/m9.figshare.28585139.
